# Cognitive impairments associated with CFS and POTS

**DOI:** 10.3389/fphys.2013.00113

**Published:** 2013-05-16

**Authors:** Lindzi Shanks, Leonard A. Jason, Meredyth Evans, Abigail Brown

**Affiliations:** Center for Community Research, DePaul UniversityChicago, IL, USA

**Keywords:** chronic fatigue syndrome, fatigue, POTS, cognitive impairments

## Abstract

Chronic fatigue syndrome (CFS) is characterized by fatigue, sleep dysfunction, and cognitive deficits (Fukuda et al., 1994). Research surrounding cognitive functioning among patients with CFS has found difficulty with memory, attention, and information processing. A similar disorder, postural tachycardia syndrome (POTS), is characterized by increased heart rate, fatigue, and mental cloudiness (Raj et al., 2009). Potential implications of cognitive deficits for patients with CFS and/or POTS are discussed, including difficulties with school and/or employment. A few biological theories (i.e., kindling, impairments in the central nervous system, and difficulty with blood flow) have emerged as potential explanations for the cognitive deficits reported in both CFS and POTS Future research should continue to examine possible explanations for cognitive impairments in CFS and POTS, and ultimately use this information to try and reduce cognitive impairments for these patients.

Myalgic Encephalomyelitis/chronic fatigue syndrome (CFS) is an illness characterized by persistent excessive fatigue (i.e., tired), post-exertional malaise, flu-like symptoms, and cognitive impairments (Fukuda et al., 1994). Diagnostic criteria varies due to a poor understanding of the illness. However, patients with CFS are often diagnosed after experiencing debilitating fatigue for at least 6 months and the presence of fatigue cannot be explained by any other medical condition. Sleep and rest often do not help with energy levels (Fukuda et al., 1994). Cognitive impairments in individuals with CFS involve problems with visual memory (Grafman et al., 1993), verbal memory (DeLuca et al., 1995; Joyce et al., 1996), attention span (McDonald et al., 1993), reaction times (Prasher et al., 1990), concentration (Krupp et al., 1994), and complex information processing (DeLuca et al., 1993, 1995). However, the literature is inconsistent regarding these cognitive impairments (Altay et al., 1990; Scheffers et al., 1992; Johnson et al., 1994). Despite conflicting findings, patients with CFS subjectively report difficulties with cognitive functioning (Vercoulen et al., 1994; Tiersky et al., 1997), and they experience cognitive impairments in their daily lives.

Similarly, postural tachycardia syndrome (POTS) is a disease characterized by orthostatic tachycardia without orthostatic hypotension. In other words, patients with POTS often experience an excessive increase in heart rate when they stand up. POTS is known to be comorbid with CFS. In particular, POTS is often commonly found to be comorbid with adolescents with CFS (Stewart et al., 1999). Diagnostic criteria for POTS includes a heart rate increase greater than 30 beats per minute when patients go from laying down to standing. Symptoms should last more than 6 months, and patients should not experience other causes of orthostatic symptoms or tachycardia such as acute dehydration. POTS is treated in a number of ways including changing the patient's diet, increasing exercise, and medication such as beta-blockers (Abed et al., 2012).

Patients with POTS often complain of symptoms such as tachycardia, intolerance to exercise, lightheadedness, fatigue, painful headaches, and mental clouding. This mental clouding is often referred to as “brain fog” (Raj, 2006). Brain fog has been a difficult and perplexing phenomenon to explain. It may also be present in individuals with CFS who report problems with memory and inattention. The literature surrounding POTS related cognitive impairment is scarce, but the existing studies suggest difficulties with memory and attention (Raj et al., 2009). Thus, more research on cognitive deficits in POTS is necessary to better understand the implications of this disorder on cognitive functioning.

It is probable that reported cognitive impairments in both CFS and POTS would negatively impact social and daily functioning, especially in terms of occupational and educational responsibilities. Thus, cognitive impairments may decrease quality of life for patients. The present article aims to review the cognitive impairments associated with CFS and POTS, as well as cognitive impairment's potential implications on social functioning. Both CFS and POTS are included in this article because of the similarities and typical comorbidity of these two disorders. Finally, potential explanations for the existence of cognitive impairments in patients with CFS and/or POTS are examined.

## Cognitive impairments in CFS

### Memory impairments

Patients with CFS often report problems with memory. DeLuca et al. (1997) explored memory in individuals with CFS compared to a healthy control group. They found individuals with CFS performed significantly worse on cognitive tests that examine immediate and delayed visual retention. Additionally, they performed significantly worse on the short and long delay free recall tasks, which assess verbal memory. Such performance indicates memory difficulties and deficiencies among patients with CFS.

Other researchers have suggested that memory impairments may be attributed to the presence of another condition, such as major depression, rather than solely to CFS (Coyle et al., 1994). The argument that depression could play a huge role in cognitive impairments among patients with CFS is understandable since previous research estimates that up to 80% of CFS patients may concurrently have depression (Ax et al., 2001; Taillefer et al., 2002, 2003). However, DeLuca and colleagues (1997) found cognitive impairments in patients with CFS could not be explained by the presence of a depressive disorder. When compared to patients with major depressive disorder, patients with CFS performed significantly worse on working memory and free recall tasks. The same study obtained measures of depression in patients with CFS and found no correlation between depressive measures and performance on cognitive tasks (Constant et al., 2011). The cognitive tasks administered include two working memory tasks. The first was the working memory task, TEA1.5 battery, which has the participant press a button when a given stimulus (a digit which appears in the center of the screen) is identical to a previously show stimulus. The second working memory task was the computerized paced auditory serial attention test (PASAT) This task presents single digits in an auditory manner. Subjects add each new digit to the one immediately prior to it (Constant et al., 2011). Therefore, it seems patients with CFS may experience memory deficits with or without comorbid depression. Other studies have suggested that the appearance of memory difficulties in patients with CFS is the result of impairments with information processing (Cockshell and Mathias, 2013) rather than actual memory deficits.

### Attention impairments

As with memory, findings regarding attention impairments in patients with CFS have been inconsistent. Research regarding attention impairments in patients with CFS focuses on sustained attention abilities, attentional-shift problems, and problems with divided attention. Patients with CFS tend to perform poorly on a continuous performance task, which measures attention abilities and reaction time (Lutgendorf et al., 1995). Another study by Joyce et al. (1996) administered an attentional set-shifting task to both participants with CFS and controls. They found participants with CFS dropped out or failed the task more frequently than controls. Patients with CFS who complain of greater mental fatigue also exhibit significant impairment on a sustained attention task when compared to controls (Capuron et al., 2006).

Patients with CFS also have problems with divided attention (Moss-Morris, 1996) and attention capacity (Marshall et al., 1997). They report more difficulties when they need to divide their attention between multiple modalities (Ross et al., 2001). Impairments in divided attention could create problems for patients with CFS in terms of occupational difficulties. For example, they might have a difficult time working on a project which requires good multi-tasking abilities.

Similarly, children with CFS experience problems with sustained and focused attention. A qualitative study conducted by Tucker et al. (2011) found parents, teachers, and children frequently describe problems with focused and sustained attention among children with CFS. Their reported cognitive difficulties included: problems staying on task, difficulty understanding directions, and a lack of mental stamina required to complete tasks. Children in the Tucker et al. (2011) study also had low scores on the Test of Everyday Attention for Children. This test is designed to assess a child's ability to divide their attention (i.e., pay attention to two things simultaneously). Results from the above-mentioned studies suggest that children with CFS have difficulty in multiple areas of attention.

### Complex information processing impairments

While some research suggests that complex information processing impairments do not exist in patients with CFS (Altay et al., 1990; Scheffers et al., 1992), there are many studies that differ with this interpretation (DeLuca et al., 1997; Tiersky et al., 2003). Difficulties with information processing, for example, include difficulty processing new information on top of existing information. It is possible that impairments in complex information processing could be the root of other cognitive problems experienced by patients with CFS (DeLuca et al., 1993). DeLuca and colleagues (2004) found that when compared to healthy controls, patients with CFS were impaired on information processing speed. In other words, patients with CFS are unable to process information at a normal rate, but rather it takes them much longer to process information. Three different literature reviews (Tiersky et al., 1997; Michiels and Cluydts, 2001; Cockshell and Mathias, 2010) examined measures of both simple (reaction time tasks) and complex (Paced Auditory Serial Addition Test) information processing speed. They found moderate to large information processing impairments in patients with CFS. Overall, this finding demonstrates that patients with CFS may have difficulty processing new and/or complex information. Therefore, patients with CFS may not have difficulty with memory *per se*, but rather with information is presented to patients with CFS too quickly the information is unlikely to be processed. In fact, Cockshell and Mathias (2013) found that when compared to healthy controls, patients with CFS showed impaired information processing speed, yet demonstrated similar performance on tests of attention, memory, motor functioning, verbal ability, and visuospatial ability. Again, this finding demonstrates that cognitive dysfunction among patients with CFS is related to impaired information processing rather than memory difficulties.

### Implications for daily functioning

Complaints of cognitive problems have been found to occur in 85–95% of individuals with CFS (Komaroff and Buchwald, 1991; Grafman, 1994). Thus, for the majority of patients with CFS, problems with memory, attention/concentration, and information processing have direct implications for daily functioning. Additionally, the degree of cognitive impairment among patients with CFS has been found to correlate with their degree of functional impairment (Christodoulou et al., 1998). Cognitive problems also affect other aspects of everyday life (Tiersky et al., 2001). Cognitive impairments could contribute to problems in education/school, employment, and social interactions.

From an educational perspective, children with this illness could have difficulties in school which could potentially lead to poor grades or being held back a grade. A qualitative study of patients with CFS found patients often feel as though their learning abilities have deteriorated (Soderlund et al., 2000). This could be a result of cognitive impairments. Rangel et al. (2000) found that half of the study's participating children with CFS were bedridden and some were confined to wheelchairs at some point during their illness. Two-thirds of the children were unable to attend school, with an average time absent from school of 1 year. When the illness was at its worst, the average student missed 17 months of school. A physical inability to attend school combined with difficulty with memory and attention could lead to significant academic struggles for children and adolescents with CFS.

Difficulties with cognitive impairments could also lead to problems with employment. Tiersky and colleagues (2001) conducted a longitudinal study of patients with CFS to examine how various aspects of their disorder change over time, including depression, employment status, and cognitive abilities. They found that although over time patients with CFS did improve slightly on cognitive functioning, they continued to have problems with employment. At follow-up, 68% were unemployed. It is possible that problems with memory, attention, and information processing create an employment environment that becomes too strenuous and frustrating to maintain. Presumably, since individuals with CFS have impairments that indicate slower cognitive functioning, it is possible they are not always able to perform cognitive tasks at a speed required or desired by an employer. This could explain why patients with CFS have problems finding or maintaining employment in addition to difficulty with physical symptoms (i.e., fatigue, malaise, etc.).

## Cognitive impairments in POTS

### Attention/concentration impairments

Problems with attention are also found in patients with POTS (Raj et al., 2009). A case study of a 16-year-old boy with POTS describes his difficulties with attention. He had to stay at home instead of attending school because he was having such persistent problems with attention and concentration with his work (Fischer et al., 2010). Raj and colleagues (2009) administered the inattention subtest from the Connors Adult ADHD Rating Scale and found patients with POTS scored significantly higher than the control subjects. These results may indicate that patients with POTS have more diminished attention and concentration difficulties when compared to healthy controls.

Difficulty with attention and concentration is often described by the term “brain fog” (Raj et al., 2009). Brain fog is a type of mental clouding characterized by lightheadedness and headaches (Abed et al., 2012). Research describing brain fog in POTS is scarce. However, the term brain fog has also been used by patients with CFS to describe their cognitive difficulties (Allen, 2008). Patients with POTS often describe their brain fog using words such as “confusing,” “cloudy,” and “easily distracted.” They describe problems associated with brain fog including “dizziness,” “fatigue,” and “lightheaded” (A. Ross, pers. communication, July 11, 2012). In other words, the mental clouding (brain fog) described by patients with POTS could be similar to the memory and attention problems experienced by patients with CFS, but is simply under-researched using a POTS population. Further research aimed at quantifying and describing the phenomenon known as “brain fog” is necessary in order to fully understand cognitive difficulties experienced by patients with POTS.

### Working memory impairments

Recent research has begun to examine neurocognitive impairments in patients with POTS, primarily difficulty with working memory and information processing. In order to examine the relationship between cognitive dysfunction and POTS, Stewart et al. (2012) had participants complete and N-back task while on a tilt-table. A tilt-table task involves having the participant lie on a bed that is tilted upward, which simulates standing. The test is designed to increase orthostatic stress. An N-back task in a cognitive test designed to asses working memory and information processing ability. The task involves showing participants a letter of the alphabet and having them say if the same letter was presented before a certain number of times back. In other words, if a participant is in a 2-back portion of the task, they would be shown three letters at separate times, for example, Z then D then Z. They would be asked if the current letter, Z, is the same as the letter presented 2-back. In this case, the answer is yes because Z was also presented two letters back. Now, if they were in a 2-back and were shown Z then D then Q, then answer is no because Q was not shown 2-back, but rather Z was shown. This is done for up to four letters back. Stewart et al. (2012) found increasing orthostatic stress in patients with CFS/POTS was related to poor performance on the N-back task. This demonstrates neurocognitive impairment for patients with CFS/POTS.

### Implications for daily functioning

Research examining difficulties with daily and social functioning for patients with POTS has been limited to the relationship between physical impairments and daily functioning (Benrud-Larson et al., 2002). Thus, research examining the influence of cognitive difficulties on patient functioning is almost non-existent, with the research that does exist being limited to case studies of attention difficulties in children with POTS (Fischer et al., 2010). We can infer based on findings related to impairments in patients with CFS that problems with attention and concentration for patients with POTS could lead to difficulties with school for adolescents. This could include problems with staying on task, attendance, and difficulty processing information. Additionally, problems with attention and memory in POTS patients could lead to problems with employment. More research is needed.

## Cognitive impairment theories

One of the cardinal symptoms of CFS is cognitive impairment (Fukuda et al., 1994), and a large number of patients with CFS complain of difficulties with cognitive functioning (Komaroff and Buchwald, 1991; Grafman, 1994). However, it is unclear what underlying mechanism(s) are behind the cognitive impairments reported in CFS. Recent research has attempted to explain the biological mechanisms of CFS and POTS that could lead to the associated symptomatology. Current theories which aim to explain cognitive difficulties among patients with CFS and/or POTS are discussed below, including kindling theory, central nervous system impairments, difficulties with blood flow, and impairment with a number of neurotransmitters.

### Kindling

One potential explanation for cognitive deficits in patients with CFS is a concept known as kindling. Kindling theory states that when neurons are repeatedly exposed to a stimulus, they can eventually reach a point of hypersensitivity. This state of hypersensitivity can induce seizure like activity (Goddard, 1967). In other words, patients with CFS might be exposed to repeated low-intensity stimulation brought on by a virus. The seizures ultimately induced by such stimulation could spread to the rest of the brain and become the root of a number of CFS symptoms (Jason et al., 2011). Kindling is a type of central nervous system impairment.

Jason and colleagues (2009) developed a scale, which includes several fatigue symptoms for patients with CFS. One of these symptoms was described as an “over stimulation of the mind or body without the available energy to act out the mental or physiological excited state.” This symptom was present for patients with CFS, but was not present among healthy individuals. It is possible that this symptom is due to kindling. If kindling is creating a mental state in which the mind is being constantly stimulated, it could lead to fatigue from constant use. Therefore, kindling in patients with CFS could cause mental fatigue, and in turn they would lack the energy necessary to complete cognitive tasks. Kindling theory could ultimately explain why patients with CFS have difficulty with memory, attention, and information processing tasks.

### Other central nervous system impairments

Another theory potentially explaining cognitive difficulties among patients with CFS focuses on impairments within the central nervous system. Both structural (Buchwald et al., 1992; Natelson et al., 1993; Cope and David, 1996; Lange et al., 1999) and functional (MacHale et al., 2000; de Lange et al., 2004) neuroimaging studies have attempted to examine some of the possible cerebral correlates of CFS symptoms. A study conducted by de Lange and colleagues (2005) found a significant decline in gray matter (GM) volume among those with CFS when compared to healthy controls. This marked reduction in GM could explain the fatigue and cognitive deficits associated with CFS, and provide evidence that CFS difficulties are due to GM impairments within the central nervous system.

Similarly, de Lange et al. (2008) found patients with CFS have reduced GM when compared to healthy controls. Participants with CFS were provided a non-pharmacological intervention and participation in this program led to significant increases in GM, localized in the lateral prefrontal cortex. This change in GM was also related to significant improvements in cognitive speed among patients with CFS. This finding provides evidence supporting the relationship between the central nervous system and cognitive impairments in patients with CFS.

### Blood flow in POTS

POTS is characterized by lightheadedness, headache, fatigue, mental cloudiness, and pooling of blood (Medow and Stewart, 2007). One theory that has emerged suggests that cognitive impairments and mental cloudiness associated with POTS is associated with cerebral blood flow. Similarly to CFS, this results from a program with the central nervous system. Substantially lowered cerebral blood flow occurs about 50% of the time in patients with POTS when compared to controls. The lower cerebral blood flow can impair cerebral perfusion and neurocognitive function (van Lieshout et al., 2003). A significant decrease in brain perfusion could potentially explain lightheadedness, dizziness, and mental cloudiness that are common among patients with POTS (Low et al., 1999; Schondorf et al., 2005). Ocon and colleagues (2009) found patients with POTS had decreased cerebral blood flow after being placed upright during tilt-table testing. Panerai and colleagues (2005) found other tests, the critical closing pressure (CCP) and resistance area product (RAP), to be better measures of cerebral blood flow responses induced by mental activation tasks rather than cerebrovascular resistance alone. Stewart et al. (2012) has found increasing orthostatic stress impairs cognitive functioning in CFS/POTS. However, these impairments are not related to cerebral blood flow using cerebrovascular resistance alone, but when CCP and RAP were used, cerebral blood flow was related to neurocognitive abilities. In other words, since patients with POTS experience lower cerebral blood flow, it could explain why they experience some cognitive impairment.

### Neurotransmitters in CFS and POTS

Additionally, there is research examining the role neurotransmitters play in cognitive impairments in patients with CFS and POTS. Previous researchers have found impaired functioning of the norepinephrine transporter (NET) contributes to orthostatic intolerance (OI), a symptom of both CFS and POTS (Jacob et al., 2000; Lambert et al., 2008; Shannon et al., 2000; Bayles et al., 2012). In contradiction, Goldstein et al. (2002) did not find any evidence of NET dysfunction. OI occurs when patients with CFS stand up quickly and become dizzy and lightheaded. If OI occurs when the norepinephrine transporter is inhibited, then impairments with the norepinephrine transporter could help explain the cognitive impairments and mental cloudiness associated among patients with CFS and POTS.

Shannon et al. (2000) found that in the case of one family, the cause of POTS was traced to a point mutation in the coding region of the NET gene which produced a dysfunctional transporter protein. This defect was found to augment the sympathoneural signal, and also increased the rate of overflow of norepinephrine into plasma. For the rest of the POTS patients, a similar loss of function mutation was not identified, and in turn, the cause of the phenotype of impaired NET activity continues to be unknown. Bayles et al. (2012) performed an analysis of polymorphisms in the NET gene in POTS patients and healthy controls. Bayles et al. (2012) also tested for an alternative epigenetic cause of NET dysfunction. When altered NET gene sequence or promoter methylation is not present, reduced NET protein expression in white blood cells was related to chromatin modifications, which indicates an epigenetic gene silencing mechanism. This could potentially be responsible for the symptoms of POTS, including cognitive impairments, among some POTS patients and comorbid CFS patients.

Other studies report CFS and POTS symptoms are directly related to abnormalities of central neurotransmitters including corticotrophin-releasing hormone (CRH) (Demitrack et al., 1991; Bakheit et al., 1992; Sharpe et al., 1996). The main function of CRH is to produce behavioral and locomotors function. Deficiency of CRH could explain chronic fatigue, and in turn, cognitive impairments associated with chronic fatigue.

It is clear that understanding cognitive impairments and symptoms in CFS and POTS is not a simple task. However, the common theme suggests some sort of impairment within the central nervous system. Further research should focus on the central nervous system as a possible gateway of understanding the etiology of these disorders further.

## General discussion

Patients with CFS and POTS suffer from cognitive impairments that could lead to difficulties with daily functioning. Although there are studies that question the presence of cognitive impairments in CFS (Altay et al., 1990; Scheffers et al., 1992), a large number of studies indicate difficulties with memory (Grafman et al., 1993), attention (McDonald et al., 1993), and information processing (DeLuca et al., 1993). CFS memory problems include difficulties with visual and verbal memory as well as problems with short-term and long-term recall (DeLuca et al., 1995; Joyce et al., 1996). Memory deficits could lead to impairments in daily functioning as well as difficulties in school and at work. Students with CFS could have difficulty with tasks such as remembering information presented in a lecture, while adults with CFS could have difficulty at work with remembering tasks assigned to them by their employer. Patients with CFS also have difficulty with concentration/attention (Krupp et al., 1994) and complex information processing (DeLuca et al., 1993, 1995). These cognitive deficits could also lead to problems with daily functioning.

Patients with POTS experience a mental clouding known as “brain fog” (Raj et al., 2009) which could lead to difficulties with attention and concentration. Children with POTS often experience more difficulty with attention in the classroom when compared to healthy children (Raj et al., 2009). This could lead to poor classroom performance for a number of reasons. Potentially, children with POTS will perform worse in school because they are unable to attend to the information presented in the classroom. Additionally, children with POTS could have poor classroom performance because a teacher might mistake their attention difficulties for a behavior problem, and thus not make the proper adjustments necessary to try and increase classroom success.

Cognitive impairments in both CFS and POTS have the potential to create problems with daily functioning, including difficulties with school and employment. Cognitive impairment is a core symptom of CFS, and the presence of cognitive impairments is part of the definition of CFS (Fukuda et al., 1994). Yet, it is currently unclear as to how such cognitive impairments are directly related to impairments in daily, occupational, educational and/or social functioning. Several potential etiological models are briefly reviewed in this paper, but more research as to how cognitive deficits and functioning are directly related is necessary to better understand the significance and cause of these symptoms. Information regarding the relationship between cognitive impairment and functioning would help enhance knowledge of these disorders for patients and clinicians, ultimately helping patients to cope with such deficits and to improve quality of life.

### Conflict of interest statement

The authors declare that the research was conducted in the absence of any commercial or financial relationships that could be construed as a potential conflict of interest.
